# An Investigation on Low Velocity Impact Response of Multilayer Sandwich Composite Structures

**DOI:** 10.1155/2013/175090

**Published:** 2013-12-23

**Authors:** S. Jedari Salami, M. Sadighi, M. Shakeri, M. Moeinfar

**Affiliations:** ^1^Department of Mechanical Engineering, Damavand Branch, Islamic Azad University, Damavand, Iran; ^2^Mechanical Engineering Department, Amirkabir University of Technology, 424 Hafez Avenue, Tehran 15875-4413, Iran

## Abstract

The effects of adding an extra layer within a sandwich panel and two different core types in top and bottom cores on low velocity impact loadings are studied experimentally in this paper. The panel includes polymer composite laminated sheets for faces and the internal laminated sheet called extra layer sheet, and two types of crushable foams are selected as the core material. Low velocity impact tests were carried out by drop hammer testing machine to the clamped multilayer sandwich panels with expanded polypropylene (EPP) and polyurethane rigid (PUR) in the top and bottom cores. Local displacement of the top core, contact force and deflection of the sandwich panel were obtained for different locations of the internal sheet; meanwhile the EPP and PUR were used in the top and bottom cores alternatively. It was found that the core material type has made significant role in improving the sandwich panel's behavior compared with the effect of extra layer location.

## 1. Introduction

Sandwich structures with composite laminated face sheets and lightweight core material are being used in several parts of aircraft, aerospace, and marine structures. Generally these structures are subjected to impacts such as tool drops, hail, bird strikes, and runway debris. Although the metal sheets are stronger than fiber reinforced plastics against static loadings but they are incapable under impact loads and humidity [1]. The impact loadings generally cause different modes of damage in the face sheets, in the core, or in the interface between the face sheets and the core, or even in all of them. As a result of these kinds of damage, the mechanical properties of the sandwich structures will be degraded considerably. The critical failure modes are (a) core buckling; (b) delamination in the impact face sheet; (c) core cracking; (d) matrix cracking; and (e) fiber breakage in the facings [2, 3].

Impact crush performance of the core in conventional single layer sandwich structures depends upon core density, cell size of the core, material properties of the core, and the panel formation. Nettles and Hodge [4] studied the failure mode of glass phenolic honeycomb core under the impact loads and revealed that core buckling and core cracking modes occurred at very low and higher energy levels, respectively. The impact damage on sandwich panels caused significant degradations in tensile, compressive, shear, and bending strengths [3, 5–8]. Mines et al. [9] investigated the failure modes of various sandwich beams and observed that top face sheet failure followed by stable core crushing was the best mode of failure for energy absorption. The face sheet core interface is the critical surface for delamination under localized impact loading and leads to rather weak impact strength [10–13]. Saraswathy et al. [14] studied dynamic response of honeycomb sandwich beam with multiple debonds. In this research, the frequency response functions are obtained by nonlinear transient analysis of frequency of CFRP sandwich beam with debond. Frequencies are obtained for two types of model having single debond and double debond at different spacing between them. In order to improve the delamination resistance of the face sheet core interface, Vaidya et al. [15] introduced an integrated hollow core sandwich panel that caused enhanced low velocity impact strength.

There are few studies on the mechanical response of the multilayer sandwich panels in the literature so far. Stickney and Abdulhadi [16] introduced a small deflection theory for the flexure of multilayer circular sandwich plates by extremizing the complementary energy with the use of Lagrange multipliers and examined the effects of shear and the degree of orthotropy on the deflection for several symmetrical and nonsymmetrical cases. Thomsen [17] proposed a general theory for the analysis of multiple layer plate assemblies consisting of interchanging high density, high stiffness layers, and low density, low stiffness/compliant interface layers and predicted the complete deformation and stress fields in a 7-layer cantilever sandwich plate composed of 4 solid laminates interfaced by 3 compliant interface layers. Suvorov and Dvorak [18] introduced and investigated a modified design that protected the foam core by inserting a ductile foam as an interlayer under the external face sheet under low velocity impact. They observed that inserting interlayer foam reduced overall and local deflections of the face sheet, local compression of the foam core, and residual stresses. Jiang and Shu [19] investigated the effects of internal sheet inserted into the honeycomb core in different locations under low velocity impact and concluded that local displacement of the core along the direction of the impact was decreased significantly by introducing the internal sheet into a traditional single sandwich structure. Bahei-El-Din and Dvorak [20] compared the behavior of conventional and modified sandwich plate designs under blast loads. The modified plate included a thin ductile interlayer as a hyperelastic PUR that was inserted between the top face sheet and the foam core. The results showed that utilizing an interlayer causes a much reduced core compression, face sheet vibration, and overall deflection compared to the conventional design. Wang [21] studied the compressive behavior of multilayer corrugated sandwich structures experimentally and concluded that compressive resistance was similar for the same type of corrugated sandwich structures with different layers; the energy absorption of the multilayer corrugated sandwich structures was significantly greater than the monolayer one and had compression resistance capability for repetitious shock. Mamalis et al. [22] used metal sheets at the outer surfaces and introduced a new concept called hybrid sandwich structure to maximize rigidity while introducing in-between lightweight cores adhesively bonded to keep the whole structure together. There are some limitations on panel thickness in structures such as in aerospace panels. A suitable way to increase the panel resistance under local loads and to decrease local deformation is to insert an internal sheet within the soft cores. In all previous studies on multilayer sandwich panels the effects of either using two different foams in top and bottom cores or inserting internal composite laminated face sheet in various locations through the thickness of core were investigated. The aim of this study is to consider both parameters on local displacement of top core, contact force, and deflection of sandwich panels under low velocity impact loads.

In this research, by inserting an additional sheet called the internal sheet, to separate top and bottom core, a multilayer sandwich structure is created. The material of each top and bottom cores could also be different. The low velocity impact tests that were applied are extended to clamped boundary condition on a multilayer sandwich panel and another type of foam core, expanded polypropylene (EPP). The crushable expanded polypropylene (EPP) and PUR foams are used in the top and bottom cores alternatively in the targets. In this regard, the effects of various cases are studied. The cases are as follows: (1) both top and bottom cores are EPP; (2) top core is EPP and bottom core is PUR; (3) top core is PUR and bottom core is EPP; (4) both top and bottom cores are PUR. Also, in each case, the influences of the location of internal sheet through the thickness of structure are investigated. The purpose of introducing this structure is to decrease the local displacement of top core, peak contact force, and deflection of the sandwich panel under impact point. The peak contact force and local displacement have an important role in development of the local crush and buckling of the core under the impact point. Thus, as it would be discussed in the present paper, it is possible to reduce damaging factors such as peak contact force and local displacement by introducing multilayer sandwich panels.

## 2. Experimental Investigation

### 2.1. Specimens and Material Properties

The material properties of the top face sheet, bottom face sheet, and internal face sheet are E-glass fiber reinforced epoxy resin matrix composite laminate. The mechanical properties of composite laminated sheets are determined using ASTM (american standard test methods) D638 M; see Table 1. A description of ply sequences and dimensions of laminates are explained in Table 2. The ply sequences and thickness of the top and bottom face sheets are the same but differ from the internal sheet. At first, fibrous composite was made by hand lay-up with various layer arrangements and then bonded to foam cores by the same epoxy resin as it was applied for laminating. Hand lay-up method pursued by curing process includes pressurizing to reduce voids and removal of excess resin. Also, the mechanical properties of foams which were used in quasi-static and low velocity impact tests are shown in Table 3.

**Figure 1:**
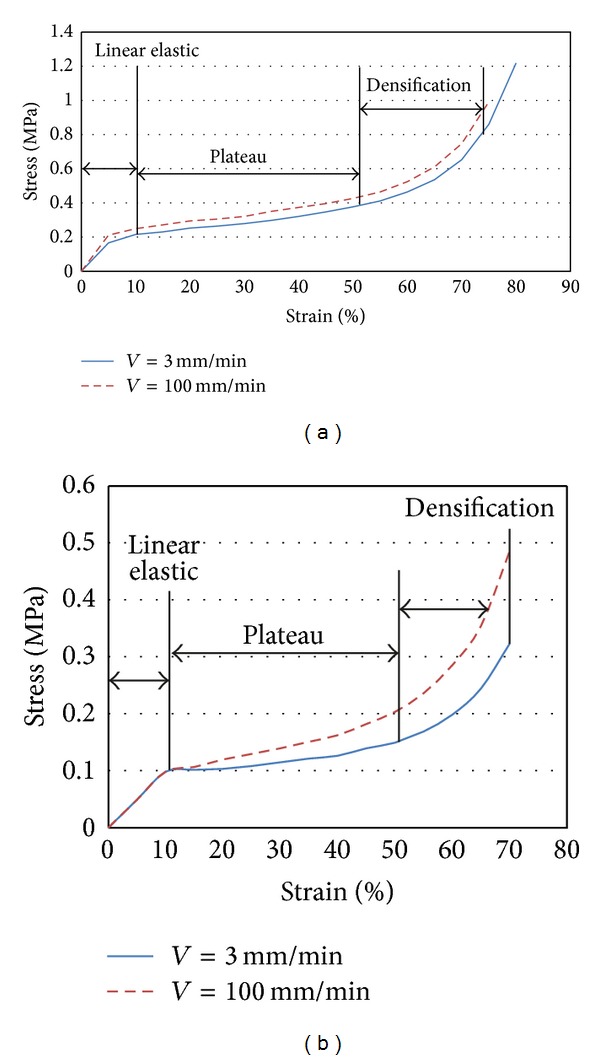
Stress-strain curves of crushable foams under quasi-static loadings in two rates: (a) EPP; (b) PUR.

As EPP and PUR foams were selected for the low velocity investigation, the specimens of these foams selected according to ASTM, D1621/94 code, were tested under quasi-static loading in two rates (3 and 100 mm/min) and the stress-strain data of them are shown in Figure 1. According to the results these kinds of foams are not considerably sensitive to strain rate. The stress-strain curves include overall three regions of stress-strain curves of crushable foams including linear elastic, plateau, and densification.

### 2.2. Low Velocity Impact Tests

**Figure 2:**
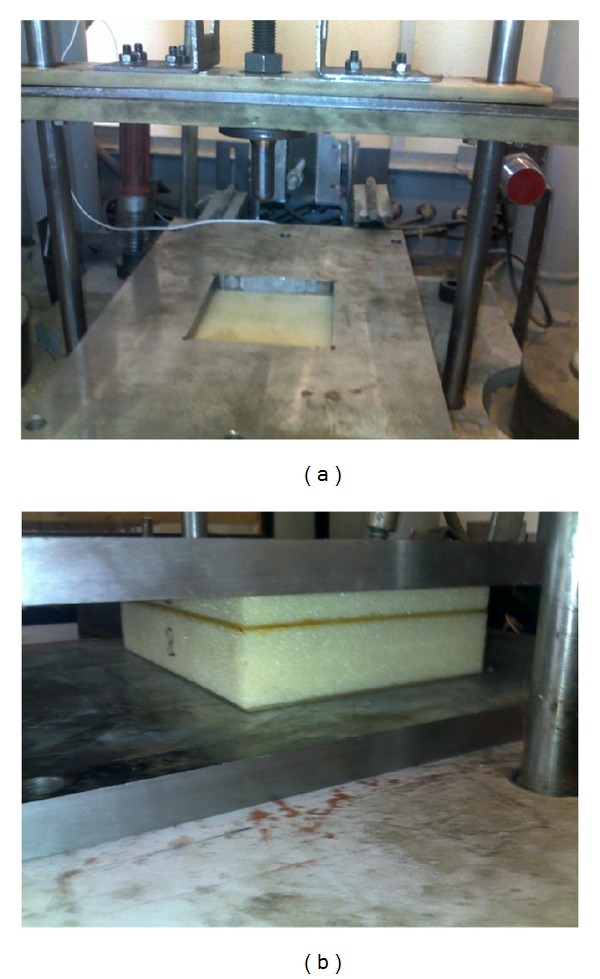
Description of the impact on a multilayer sandwich structure by the drop hammer testing machine.

**Figure 3:**
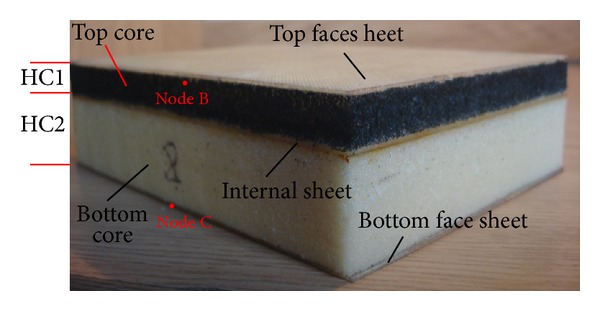
The multilayer sandwich specimen with two different core types in top and bottom cores.

The low velocity impact tests were performed using the drop hammer testing machine; see Figure 2. In all tests, the projectile is a hemispherical steel tip with diameter of 6.35 mm and the mass and contact velocity of the projectile are 0.5 kg and 3 m/s, respectively. So, the impact energy is kept at 2.25 J in all cases. The multilayer sandwich panel includes five components. They are specified from top to bottom of panel as the top face sheet, top core, internal sheet, bottom core, and bottom face sheet, respectively. The total thickness of the core is maintained at 25 mm in all cases and thickness of the top core is specified by HC1 as shown in Figure 3. In order to study the influences of the location of internal sheet through the thickness of structure for each case, three locations of internal sheet are considered. They are assumed as HC1 = 3 mm, HC1 = 6 mm, and HC1 = 12 mm. In each case the results are compared with the conventional one (without internal sheet, HC = 25 mm).

In order to analyze the local displacement of the top core during the impact, a node under the point of impact in the top core is selected. The displacement of Node B characterizes the local deformation of the element of the top core during impact. Since the displacement of bottom sheet represents the whole deflection of the target, a node is determined in the bottom sheet marked as Node C. So the displacement of this node describes the deflection of the entire target. Signals obtained by displacement sensor which are connected with nodes B and C are output to the data acquiring and transmitting device.

## 3. Results and Discussion

To evaluate the effects of putting two different types of crushable foams, EPP and PUR, in the top and bottom cores on the behavior of sandwich panel under low velocity impact, investigations were performed in four cases. The cases are as follows: (1) both top and bottom cores are EPP (EPP-EPP); (2) top core is EPP and bottom core is PUR (EPP-PUR); (3) top core is PUR and bottom core is EPP (PUR-EPP); (4) both top and bottom cores are PUR (PUR-PUR). Also, in order to study the influences of the location of internal sheet through the thickness of structure, in each case, the three locations of internal sheet are considered and marked as HC1 = 3 mm, 6 mm, and 12 mm. The results of these locations are compared with conventional panel (HC1 = 25 mm) in Cases (1) and (4). In order to represent smoothed results, in all of collected data, the dispersed data are refined.

### 3.1. Contact Forces

**Figure 4:**
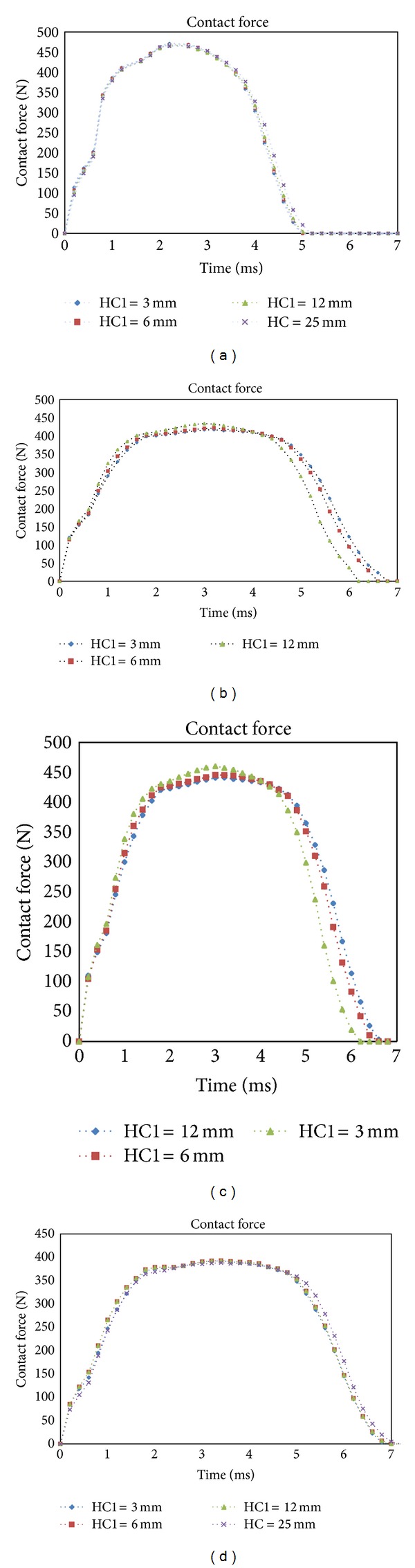
Contact forces of the different locations of internal sheet: (a) Case 1: EPP-EPP; (b) Case 2: EPP-PUR; (c) Case 3: PUR-EPP; (d) Case 4: PUR-PUR.

In order to investigate onset of failure, study on contact force between the projectile and the target is an essential parameter. The contact forces for multilayer sandwich panel in four cases and different locations of internal sheet are shown in Figure 4. In Cases (1) and (4), it could be observed that there is no significant difference among peak contact forces with varying of the location of internal sheet. This is due to the fact that compressive stiffness of target depends on material property mainly and since the foams of the top and bottom cores are the same in these two cases, the peak contact force increases slightly by decreasing HC1 due to the effect of much more stiffness of internal sheet. In contrast, the peak contact forces varied considerably in different location of internal sheet in Cases (2) and (3). As it was mentioned, the EPP has more stiffness than the PUR; see Table 1. By increasing the EPP material contribution in both the top and bottom cores, the stiffness of whole target is enhanced. As a result of this, it could be seen that in Case (2), since the EPP is put in the top core, the peak contact force is decreased by reducing the HC1 because the contribution of the EPP for the whole thickness of the core is decreased. But in Case (3), as the the EPP is inserted in the bottom core, decreasing the HC1 increases the peak contact force because the EPP contribution to the whole thickness of the core is increased.

**Figure 5:**
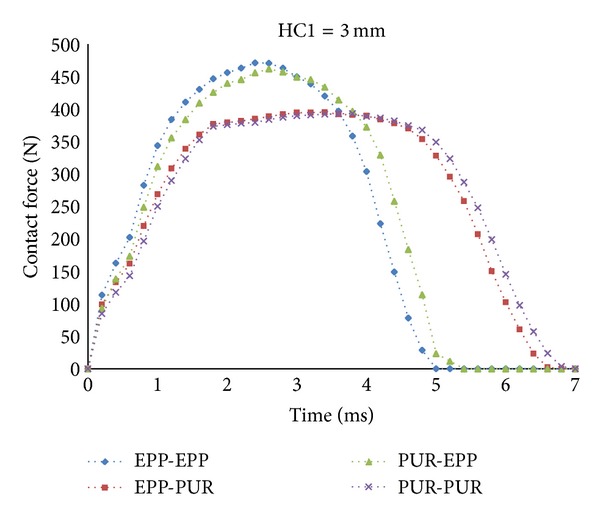
Contact forces of the target with HC1 = 3 mm in four cases of target.

For the same location of internal sheet, that is, the location corresponding to HC1 = 3 mm, as shown in Figure 5, it could be concluded that increasing the EPP contribution to the whole thickness of the core caused the peak contact force to increase and the contact time to decrease. So the peak contact force of Case (1) is the highest among other cases and the lowest one corresponds to Case (4).

### 3.2. Local Displacement of the Core

**Figure 6:**
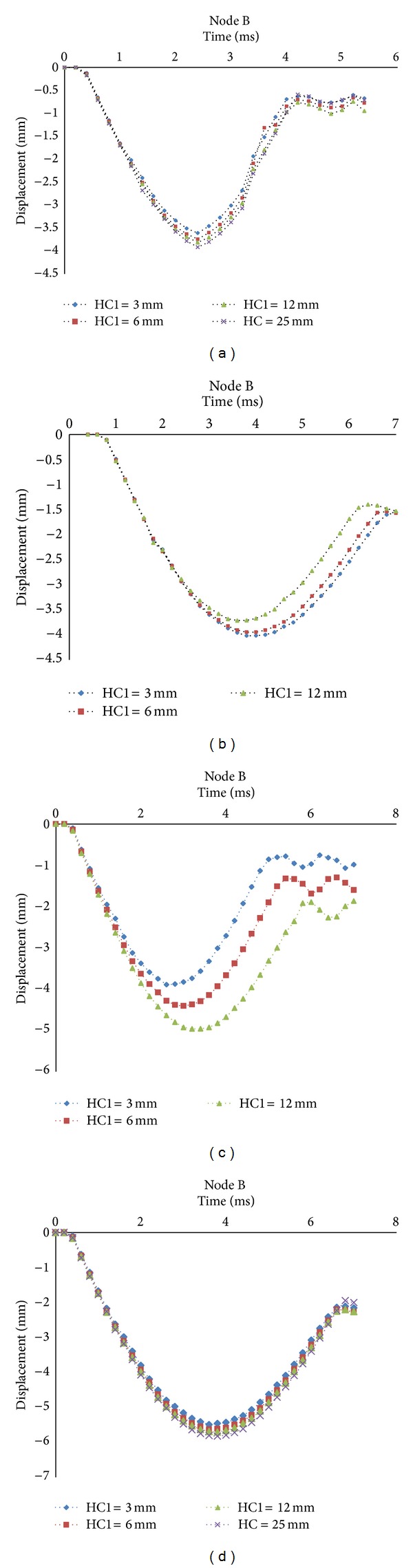
Local displacement at Node B in different locations of internal sheet: (a) Case 1: EPP-EPP; (b) Case 2: EPP-PUR; (c) Case 3: PUR-EPP; (d) Case 4: PUR-PUR.

**Figure 7:**
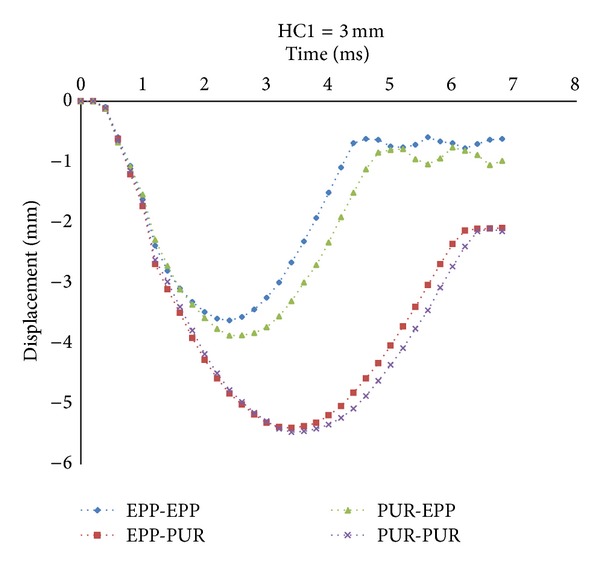
Local displacement at Node B with HC1 = 3 mm in four cases of target.

Decreasing local displacement prevents any damages and crashes in sandwich panels thanks to introducing the concept of inserting an internal sheet into the sandwiches. According to the previous sections, the displacement of Node B represents the local displacement of the core. The results for Cases (1) and (4), as shown in Figure 6, indicate that by reducing the HC1, the peak displacement of the node is decreased considerably. Since the stiffness of internal sheet is much more than core material, whenever the internal sheet is close to the top face sheet, the effect of its stiffness on reducing the local displacement of the core is more prominent. The results of Cases (2) and (3) show the influences of the top and bottom core materials on local displacement. It could be observed that by increasing HC1 the peak displacement of Node B is decreased by about 8% in Case (2). This is because that the higher HC1 is, the higher contribution of EPP is. Therefore, the compressive stiffness of target will be enhanced and it causes decrease the displacement of the top face sheet. But the results in Case (3) are the reverse of Case (2) because the bottom core is EPP and by decreasing HC1, the EPP thickness and compressive stiffness are increased. Therefore, by decreasing HC1 the peak displacement of Node B is decreased by about 22% in Case (3). In order to investigate the effects of foam type in the top and bottom cores on the local displacement of Node B, the location of internal sheet is kept identical, HC1 = 3 mm, for four cases, as shown in Figure 7. The results indicate that the peak displacement in Case (1) is the lowest and whenever the EPP contribution to the whole thickness of core is increased, the peak displacement is decreased significantly. So the peak displacement of Node B is decreased by about 33% in Case (1).

### 3.3. Global Deflection of Multilayer Sandwich Panel

**Figure 8:**
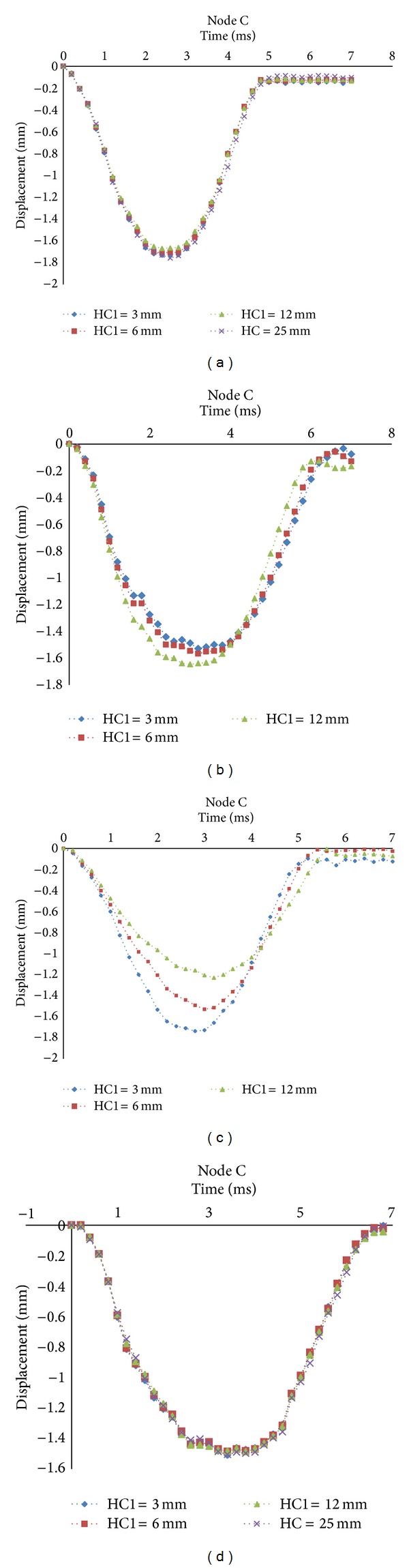
Deflection of target in different locations of the internal sheet: (a) Case 1: EPP-EPP; (b) Case 2: EPP-PUR; (c) Case 3: PUR-EPP; (d) Case 4: PUR-PUR.

**Figure 9:**
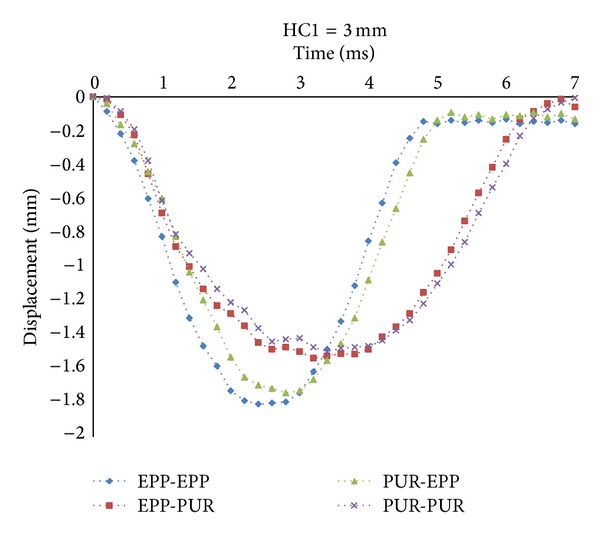
Deflection of target with HC1 = 3 mm in four cases.

The deflection of a target is determined by the displacement of a node placed at the central point of the bottom face sheet, named as Node C, as shown in Figure 3. The deflections of four cases in different locations of internal sheet are depicted in Figure 8. The results show that the location of internal sheet has no significant effects on the deflection of the target in Cases (1) and (4). The authors concluded in the previous researches that the compression profiles of EPP and PUR under low velocity impact are such that the displacement of layers through the thickness of EPP is almost the same but gradually reduced at lower layers in PUR [23, 24]. So the deflection of target is increased by increasing the EPP contribution to the whole thickness of the core, as shown in Cases (2) and (3). This is because the EPP causes the displacement of upper layers to be transferred to the bottom face sheet approximately. Thus, whenever the PUR contribution to the whole thickness of core increases, the bottom face sheet displacement is decreased. Also this occurrence is observed, while the location of internal sheet is kept fixed, as shown in Figure 9, no matter whether the locations of EPP and PUR are in the top or bottom cores.

## 4. Conclusion

In this study, a multilayer sandwich panel is investigated in order to enhance important parameters such as local displacement of core, contact force, and deflection of sandwich targets under low velocity impact. The sandwich panel consists of top and bottom cores and an internal composite laminate sheet that separates the two cores. Results of low velocity impact tests indicated that the type of crushable foam core has an important role in improving the local displacement, contact force, and deflection of sandwich panels under impact loading; that is, by increasing the compressive stiffness of foam, the whole stiffness of target is enhanced and this elevates the peak contact force and decreases local displacement of the top core. Since the compressive stiffness of EPP is more than the stiffness of PUR, by increasing the EPP contribution in the entire thickness of the core, no matter whether the location of it is in the top or bottom cores, the peak contact force is increased and corresponding local displacement of core is reduced. Also it could be concluded that whenever the internal sheet is located closer to the top face sheet, it causes a slight influence in increasing and decreasing of peak contact force and local displacement of core, respectively. On the other hand, in the compression, since EPP has more stiffness than PUR, the layers of EPP through the thickness have the same displacement, whereas the compression of layers of PUR is reduced from the upper to lower ones. Therefore, using EPP in the core transfers a significant part of the top face sheet displacement to the bottom face sheet. As a result of this phenomenon, by increasing the PUR contribution to the entire core thickness, the deflection of target is decreased significantly, no matter whether the location of it is in the top or bottom cores. Also by using a multilayer sandwich panel, it could be possible to improve local displacement of core and contact force between projectile and top face sheet with the use of stiffer crushable foams such as EPP and reduce the deflection of target by applying softer cores such as PUR.

## Figures and Tables

**Table 1:** Properties of the composite laminated sheet.

Materials	Density (kg/m^3^)	Young's modulus (Gpa)	Shear modulus (Gpa)	Poisson's ratio
Laminates	1100	*E*_*x*_ = 50,	*G*_*xy*_ = 5.43,	*ν*_*xy*_ = 0.163,
		*E*_*y*_ = 50,	*G*_*yz*_ = 3.26,	*ν*_*yz*_ = 0.0458,
		*E*_*z*_ = 9.5	*G*_*xz*_ = 5.43	*ν*_*xz*_ = 0.0263

**Table 2:** Geometrical properties of composite laminated sheet.

Sheet	Top	Internal	Bottom
Ply sequence	[0/45/90/−45/0]_*s*_	[0/90/90/0]_*s*_	[0/45/90/−45/0]_*s*_
Thickness (mm)	1.25	1	1.25
Dimensions of the target in *x-y* plane (mm)	76.4 × 76.4		

**Table 3:** Properties of crushable foams.

Core materials	Density (kg/m^3^)	Young's modulus (Gpa)	Shear modulus (Gpa)	Poisson's ratio
PUR	76	0.0011	0.00042	0.3
EPP	52	0.00367	0.00141	0.3
